# HDAC6 Inhibition Promotes Transcription Factor EB Activation and Is Protective in Experimental Kidney Disease

**DOI:** 10.3389/fphar.2018.00034

**Published:** 2018-02-01

**Authors:** Angela S. Brijmohan, Sri N. Batchu, Syamantak Majumder, Tamadher A. Alghamdi, Karina Thieme, Sarah McGaugh, Youan Liu, Suzanne L. Advani, Bridgit B. Bowskill, M. Golam Kabir, Laurette Geldenhuys, Ferhan S. Siddiqi, Andrew Advani

**Affiliations:** ^1^Keenan Research Centre for Biomedical Science and Li Ka Shing Knowledge Institute, St. Michael’s Hospital, Toronto, ON, Canada; ^2^Department of Pathology, Dalhousie University, Halifax, NS, Canada; ^3^Department of Medicine, Dalhousie University, Halifax, NS, Canada

**Keywords:** chronic kidney disease, diabetic nephropathy, autophagy, endoplasmic reticulum stress, renal tubule, Tubastatin A, histone deacetylase 6, transcription factor EB

## Abstract

To contend with the deleterious effects of accumulating misfolded protein aggregates or damaged organelles cells rely on a system of quality control processes, among them the autophagy-lysosome pathway. This pathway is itself controlled by a master regulator transcription factor termed transcription factor EB (TFEB). When TFEB localizes to the cell nucleus it promotes the expression of a number of genes involved in protein clearance. Here, we set out to determine (1) whether TFEB expression is altered in chronic kidney disease (CKD); (2) whether inhibition of the cytosolic deacetylase histone deacetylase 6 (HDAC6) affects TFEB acetylation and nuclear localization; and (3) whether HDAC6 inhibition, in turn, alters the natural history of experimental CKD. TFEB mRNA and protein levels were observed to be diminished in the kidneys of humans with diabetic kidney disease, accompanied by accumulation of the protein aggregate adaptor protein p62 in tubule epithelial cells. In cultured NRK-52E cells, HDAC6 inhibition with the small molecule inhibitor Tubastatin A acetylated TFEB, increasing TFEB localization to the nucleus and attenuating cell death. In a rat model of CKD, Tubastatin A prevented the accumulation of misfolded protein aggregates in tubule epithelial cells, attenuated proteinuria progression, limited tubule cell death and diminished tubulointerstitial collagenous matrix deposition. These findings point to the common occurrence of dysregulated quality control processes in CKD and they suggest that TFEB downregulation may contribute to tubule injury in CKD. They also identify a regulatory relationship between HDAC6 and TFEB. HDAC6 inhibitors and TFEB activators both warrant further investigation as treatments for CKD.

## Introduction

Protein misfolding is a common event. Even under normal conditions it affects around 30% of all newly synthesized proteins (Schubert et al., 2000) and it is even more common during times of cellular stress (Houck et al., 2012). When newly synthesized proteins assemble incorrectly they can accumulate within the lumen of the endoplasmic reticulum (ER). The cellular response that this elicits is termed ER stress (Chambers and Marciniak, 2014) and this is a common occurrence in chronic kidney disease (CKD) (Taniguchi and Yoshida, 2015). In some conditions, misfolded proteins can also aggregate in the cytosol and impair cellular function by obstructing signaling, disrupting mitochondrial function and interfering with microtubule organization and intracellular transit (Sweeney et al., 2017). These effects are most apparent during the development of neurodegenerative diseases such as Alzheimer’s disease or Parkinson’s disease. In contrast, the extent to which the cytosolic aggregation of misfolded proteins affects the development of CKD has been uncertain.

To counteract the deleterious consequences of protein aggregation, cells have evolved a complex set of quality control processes that dispose of misfolded proteins. These processes include the unfolded protein response (UPR), the ubiquitin proteasome system (UPS) and the autophagy-lysosome pathway. The autophagy-lysosome pathway is commonly called into play when protein misfolding exceeds the clearance capabilities of the UPS. When this occurs, ubiquitin-tagged misfolded protein monomers aggregate through the adaptor protein sequestosome-1/p62 (p62) (Geetha et al., 2012), in preparation for their engulfment by autophagosomes and subsequent degradation upon fusion of the autophagosomes with lysosomes (Dobson, 2003; Olzmann et al., 2008). Recently, it has been discovered that the autophagy-lysosome pathway is itself under the control of a “master regulator” transcription factor called transcription factor EB (TFEB) (Sardiello et al., 2009; Settembre et al., 2011). TFEB activation was found to improve lysosomal function in cystinotic kidney cells (Rega et al., 2016), whereas we reported that overexpression of TFEB can restore glomerular cell permselectivity (Alghamdi et al., 2017). TFEB activity is regulated post-translationally by its (de)phosphorylation (Roczniak-Ferguson et al., 2012; Settembre et al., 2012) and by its (de)acetylation (Bao et al., 2016). For instance, mammalian target of rapamycin complex 1 (mTORC1) phosphorylates TFEB and sequesters it in the cytosol, limiting the movement of TFEB to the nucleus (Pena-Llopis et al., 2011). Relatively less is known about mediators involved in TFEB (de)acetylation and the effects that (de)acetylation may have on TFEB activity. One regulator of protein (de)acetylation is the cytosolic protein, histone deacetylase 6 (HDAC6). HDAC6 catalyzes the deacetylation of its protein substrates and it also has non-enzymatic actions as an adaptor protein. Through both of these actions, HDAC6 plays several different roles in both nuclear trafficking of transcription factors (Boyault et al., 2007) and the bulk clearance of protein aggregates (Lee et al., 2010). Whether HDAC6 regulates the nuclear trafficking of TFEB, however, has not previously been examined.

Here, we set out to determine whether TFEB expression is altered in human and experimental CKD and whether inhibiting HDAC6 affects TFEB activity and/or the progression of renal decline in experimental CKD. To do this, we took advantage of a rationally designed small molecule inhibitor specific for HDAC6, Tubastatin A (IC_50_ for HDAC6 0.015 μM, representing >1,000 selectivity vs. HDACs 1–11 [except HDAC8, 57-fold selectivity]) (Butler et al., 2010).

## Materials and Methods

### Human Study

Kidney tissue was examined from 12 patients with diabetic nephropathy and 12 individuals without diabetes, matched for age and sex. Tissue had been obtained at the time of nephrectomy for conventional renal carcinoma, with kidney tissue examined from unaffected regions. The study was approved by the Nova Scotia Health Authority Research Ethics Board and the Research Ethics Board of St. Michael’s Hospital. A waiver of consent was provided by the Nova Scotia Health Authority Research Ethics Board based on impracticability criteria.

### Rat Studies

In *Study 1*, male Sprague Dawley rats (Charles River, Montreal, QC, Canada) aged 8 weeks underwent subtotal (5/6) nephrectomy or sham surgery as previously described (Advani et al., 2011). Briefly, under isoflurane anesthesia, a subcapsular nephrectomy was performed to remove the right kidney and two of the three or four branches of the left kidney were selectively ligated to achieve infarction of approximately two thirds of the kidney, which was left *in situ*. Sham-operated rats underwent laparotomy with manipulation of both kidneys. Rats were followed for 7 weeks before collection of the (remnant) kidney for the study of TFEB expression and p62 immunostaining. In *Study 2*, male Sprague Dawley rats were treated with Tubastatin A HCl (30 mg/kg in 5% dextrose by thrice weekly subcutaneous injection; Medchemexpress, Monmouth Junction, NJ, United States) or vehicle for 3 weeks before the collection of kidneys for determination of α-tubulin acetylation levels by immunoblotting (as a marker of HDAC6 inhibition) and for determination of renal nuclear TFEB levels by immunofluorescence. The dosing regimen was informed by a previously published abstract that reported the treatment of mice with Tubastatin A at a dose of 30 mg/kg s.c. every 3 days (Oh et al., 2014). Its efficacy in the rat study was determined by the immunoblotting experiments in *Study 2*. In *Study 3*, male Sprague Dawley rats aged 8–10 weeks underwent sham or subtotal nephrectomy surgery as already described. Four weeks later, urine protein excretion was determined after housing rats individually in metabolic cages for 24 h. Rats were then stratified to receive either Tubastatin A (30 mg/kg thrice weekly) or vehicle for a further 3 weeks. Systolic blood pressure was determined by tail cuff plethysmography (Powerlab, ADInstruments, Colorado Springs, CO, United States), as previously described (Advani et al., 2007). Glomerular filtration rate (GFR) was determined by single-shot FITC-inulin clearance and repeated sampling via the tail-vein as previously described (Advani et al., 2011). All rodent experimental procedures adhered to the guidelines of the Canadian Council on Animal Care and were approved by the St. Michael’s Hospital Animal Care Committee, Toronto, ON, Canada.

### Cell Culture Studies

For determination of α-tubulin acetylation and nuclear TFEB, NRK-52E cells (Advani et al., 2009) were treated with Tubastatin A in 0.1% DMSO for 24 h. For determination of acetylated TFEB levels, NRK-52E cells were treated with 2.5 μM Tubastatin A (Butler et al., 2010) (or vehicle) for 24 h prior to immunoprecipitation for TFEB (Abcam, Cambridge, MA, United States). For determination of ER stress in NRK-52E cells, cells were treated with 500 nM thapsigargin (Sigma–Aldrich, Oakville, ON, Canada) for 24 h. For determination of programmed cell death, NRK-52E cells were pre-treated with 2.5 μM Tubastatin A (or vehicle) for 4 h, before the addition of 500 nM thapsigargin for a further 24 h. For annexin V labeling, following labeling with PE-annexin V 7-aminoactinomycin D (7-AAD) (BD Biosciences, San Jose, CA, United States) cellular fluorescence was assessed on a MACSQuant Analyzer (Miltenyi Biotec, Cambridge, MA, United States). For gene knockdown, cells were transfected with sequence-specific siRNA or scrambled siRNA (Thermo Fisher Scientific, Rockford, IL, United States) at concentrations of 75 nM for HDAC6 and 50 nM for TFEB for 24 h using Lipofectamine 2000 (Thermo Fisher Scientific).

### Real-Time PCR

RNA was isolated from human kidney samples using the miRNeasy FFPE kit (Qiagen Sciences, Germantown, MD, United States), from rat kidney homogenates using the RNeasy Mini Kit (Qiagen Sciences) and from cultured cells using Trizol reagent (Thermo Fisher Scientific). Real time PCR was performed using SYBR green (Wisent Bio Products, St.-Jean-Baptiste, QC, Canada) on a ViiA7 PCR system (Thermo Fisher Scientific). Primer sequences were designed using Primer Blast^1^ and were as follows: human TFEB forward GTAGAGAATGATGCCTCCGCA, reverse CAGCCTGAGCTTGCTGTCAT; human RPL32 forward CAACATTGGTTATGGAAGCAACA, reverse TGACGTTGTG GACCAGGAACT; rat TFEB forward GCGGTCACTGAAGG ACAGAG, reverse GCAGCAAACTTGTTGCCGTA; rat RPL13a forward ATGAACACCAACCCGTCTCG, reverse GCCTCTTTTGGTCTTGTGCG; rat beclin 1 forward CTCGTCAAGGCGTCACTTCT, reverse CCTCCATTCTTTAGGCCCCG; rat GAPDH forward ATGCTGGTGCTGAGTATGTC, reverse AGTTGTCATATTCTCCGTGG. Data analysis was performed using the Applied Biosystems Comparative C_T_ method.

### Immunohistochemistry

Immunohistochemistry was performed on formalin-fixed paraffin embedded kidney sections (10% neutral buffered formalin, Thermo Fisher Scientific; Paraplast Plus, Sigma–Aldrich) with antibodies in the following concentrations: TFEB 1:75 (Abcam), p62 1:100 (Cell Signaling Technology, Danvers, MA, United States) and collagen IV 1:100 (Southern Biotech, Birmingham, AL, United States). Cortical TFEB was determined as the proportional area of positive immunostaining in 10 randomly selected cortical fields (×100 magnification) using ImageScope (Leica Microsystems Inc., Concord, ON, Canada) and represented as fold change relative to control. For quantification of p62 immunostaining, the number of tubules with cells positively immunostaining for cytosolic p62 was determined in 6-10 randomly selected cortical fields (×100 magnification) and represented as the fold change relative to control. For quantification of glomerular and tubulointerstitial collagen IV, kidney sections were scanned (Leica Microsystems Inc.) and quantified as the proportion of positive immunostaining area in 10 randomly chosen cortical fields (excluding glomeruli) or 30 glomeruli using ImageScope. Terminal deoxynucleotidyl transferase dUTP nick end labeling (TUNEL) was performed by the Pathology Research Program at Toronto General Hospital (Toronto, ON, Canada) and quantified as the number of TUNEL positive nuclei in 10 randomly selected cortical fields at a magnification of ×100. All histological analyses were performed by an investigator masked to the study groups.

### Immunoblotting

Immunoblotting was performed with antibodies in the following concentrations: p62 1:1000 (Cell Signaling Technology), ubiquitin 1:1000 (Cell Signaling Technology), phosphorylated eukaryotic initiation factor 2α (phospho-eIF2α) 1:1000 (Cell Signaling Technology), eIF2α 1:1000 (Cell Signaling Technology), acetylated α-tubulin 1:1000 (Sigma–Aldrich), α-tubulin 1:1000 (Sigma–Aldrich), TFEB 1:700 (Abcam), acetylated lysine 1:1000 (Cell Signaling Technology), cleaved caspase 3 1:1000 (Cell Signaling Technology), HDAC6 (NRK-52E cells) 1:1000 (Novus Biologicals, Oakville, ON, Canada), histone H3 1:2000 (Cell Signaling Technology), HDAC6 (rat kidneys) 1:1000 (Cell Signaling Technology), beclin 1 1:1000 (Cell Signaling Technology), autophagy-related protein 7 (ATG7) 1:1000 (Cell Signaling Technology), LC3 1:1000 (Cell Signaling Technology) and β-actin 1:10,000 (Sigma–Aldrich). Separation of cytosolic and nuclear fractions was performed by centrifugation of samples at 16,000 × *g*. Densitometry was performed using ImageJ version 1.46r (National Institutes of Health, Bethesda, MD, United States).

### Immunofluorescence Microscopy

Antibodies were prepared in the following concentrations: TFEB 1:100 (Abcam), p62 1:100 (BD Biosciences, San Jose, CA, United States) and LAMP-1 1:100 (Santa Cruz Biotechnology, Dallas, TX, United States); secondary antibodies were Alexa Fluor 555 donkey anti-goat IgG 1:100 (Abcam), Alexa Fluor 647 donkey anti-goat IgG 1:100 (Thermo Fisher Scientific), Alexa Fluor 555 donkey anti-rabbit IgG (Abcam) and Alexa Fluor 488 donkey anti-mouse IgG 1:100 (Abcam). DAPI (Cell Signaling Technology) was used at a concentration of 1:10,000. Slides were viewed on a Zeiss LSM 700 confocal microscope (Carl Zeiss Canada, Toronto, ON, Canada). TFEB nuclear translocation was assessed on Adobe Photoshop (CS4) (San Jose, CA, United States) as the number of red pixels in at least three randomly selected x630 fields, in at least three experimental replicates per condition for NRK-52E cells or in kidney sections from vehicle- (*n* = 5) or Tubastatin A- (*n* = 4) treated rats.

### Statistics

Statistical significance was determined by one-way ANOVA with a Fisher least significant difference test for comparison of multiple groups and Student *t*-test for comparison between two groups (or Mann–Whitney test for non-parametric data). Data are expressed as mean ± standard error of the mean (SEM). Statistical analyses were performed using GraphPad Prism 6 for Mac OS X (GraphPad Software Inc., San Diego, CA, United States).

## Results

### Transcription Factor EB Expression Is Decreased and Tubule p62 Immunostaining Is Increased in Human Diabetic Kidney Disease

**FIGURE 1:**
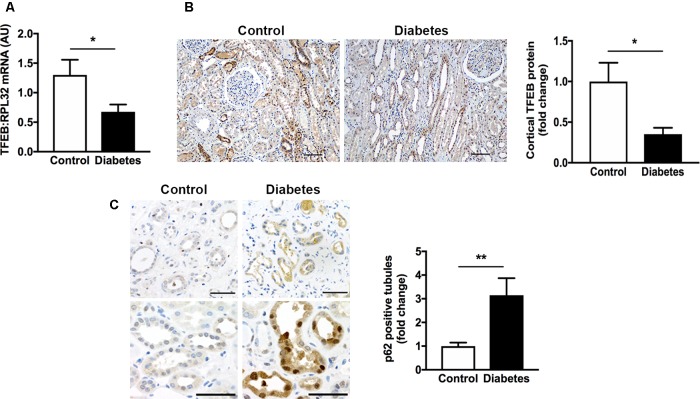
Transcription factor EB (TFEB) expression is decreased and levels of the adaptor protein p62 are increased in the kidneys of patients with diabetic kidney disease. **(A)** Real-time PCR for TFEB in formalin-fixed paraffin-embedded nephrectomy tissue from individuals with diabetic kidney disease (diabetes, *n* = 12) and individuals without diabetes and with normal kidney function (control, *n* = 12). **(B)** Immunohistochemistry for TFEB and quantification of cortical TFEB in kidney tissue from individuals with diabetic kidney disease (*n* = 7) or controls (*n* = 6). Scale bar = 100 μm. **(C)** Immunohistochemistry for p62 and quantification of tubule p62 immunostaining in kidney tissue from people with diabetic kidney disease (*n* = 10) or controls (*n* = 10). Scale bar = 50 μm. AU = arbitrary units. Values are mean ± SEM. ^∗^*P* < 0.05, ^∗∗^*P* < 0.01.

The autophagy-lysosome pathway is becoming increasingly recognized for its importance in kidney homeostasis and disease (Fougeray and Pallet, 2015) and the transcription factor, TFEB is emerging as an important regulator of the autophagy-lysosome pathway (Sardiello et al., 2009; Settembre et al., 2011). In our first experiments we addressed the question as to whether TFEB expression is altered in human CKD and whether this is associated with an accumulation of intracellular protein aggregates. We focused our attentions on the expression of TFEB in human diabetic kidney disease, the most prevalent cause of CKD. Accordingly, we examined kidney tissue from 12 individuals with histopathologically confirmed diabetic glomerulosclerosis and 12 individuals without diabetes. The clinical characteristics of the patients have been described before (Majumder et al., 2018). Using real-time PCR, we observed a ∼50% reduction in renal parenchymal TFEB mRNA levels in individuals with diabetic kidney disease in comparison to individuals without diabetes and with normal kidney function (**Figure 1A**). Paralleling the reduction in TFEB mRNA, TFEB protein levels in the tubulointerstitium were equivalently reduced in kidney sections from individuals with diabetic kidney disease compared to controls (**Figure 1B**). To assess whether decreased TFEB is associated with the presence of misfolded proteins, we immunostained kidney sections for the protein aggregate adaptor protein p62. Whereas TFEB mRNA and protein levels were diminished, immunostaining for p62 was increased approximately three-fold in tubule epithelial cells of kidney sections from people with diabetic kidney disease (**Figure 1C**), being evident in both the cytosol and the nucleus (**Figure 1C**).

### TFEB mRNA Levels Are Decreased and Misfolded Proteins Accumulate in the Kidneys of Subtotally Nephrectomized Rats

**FIGURE 2:**
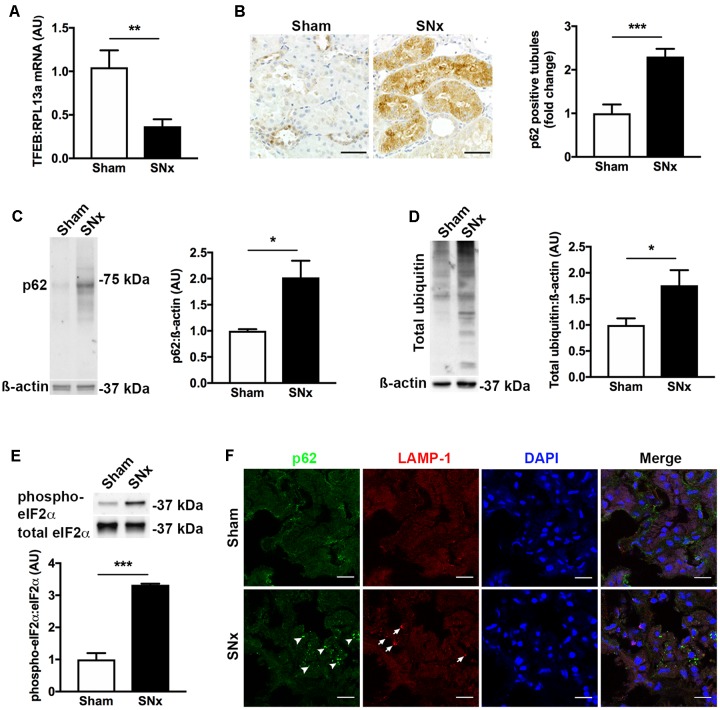
Transcription factor EB expression is decreased and misfolded proteins accumulate in the kidneys of subtotally nephrectomized (SNx) rats. **(A)** Real-time PCR for TFEB in the kidneys of sham-operated rats (*n* = 11) or SNx rats (*n* = 12), 7 weeks after surgery. **(B)** Immunohistochemistry for p62 and quantification of tubule p62 immunostaining in kidney tissue from sham (*n* = 10) and SNx (*n* = 8) rats. Scale bar = 50 μm. **(C)** Immunoblotting for p62 in kidney tissue from sham (*n* = 3) and SNx (*n* = 3) rats. **(D)** Immunoblotting for ubiquitin in kidney tissue from sham (*n* = 4) and SNx (*n* = 4) rats. **(E)** Immunoblotting for phosphorylated and total forms of eukaryotic initiation factor 2α (eIF2α) in kidney tissue from sham (*n* = 3) and SNx (*n* = 3) rats. **(F)** Dual immunofluorescence staining of kidney tissue from SNx rats showing no co-localization of p62 (arrowheads) with the lysosome marker LAMP-1 (arrows). Scale bar = 15 μm. AU = arbitrary units. Values are mean ± SEM. ^∗^*P* < 0.05, ^∗∗^*P* < 0.01, ^∗∗∗^*P* < 0.001.

To better understand the relationship between decreased TFEB expression and increased p62 immunostaining, we turned to an experimental model of CKD, the subtotally nephrectomized rat (SNx). We selected this model because, unlike most models of diabetic kidney disease, SNx rats develop GFR decline and tubulointerstitial injury (Advani et al., 2011). Similar to the changes we observed in human kidney tissue, the kidneys of SNx rats also exhibited a decrease in TFEB mRNA levels (**Figure 2A**) and an increase in the proportion of kidney tubules positively immunostaining for p62 (**Figure 2B**). To determine whether the increase in tubule p62 immunostaining was indicative of increased p62 levels or solely increased p62 visibility following aggregation, we immunoblotted kidney homogenates of SNx rats, observing an overall increase in p62 protein levels relative to sham-operated controls (**Figure 2C**). Likewise, total ubiquitin levels were also increased in the kidneys of SNx rats (**Figure 2D**) which we interpreted, together with the increase in p62 expression, as being indicative of a generalized increase in misfolded protein accumulation. This occurred in the context of approximately three-fold increase in phospho-eIF2α (**Figure 2E**), a marker of ER stress (Wang and Kaufman, 2016). Finally, to exclude the possibility that increased p62 immunostaining could be due to the presence of urinary protein-rich lysosomes in the tubule epithelial cells of SNx rats, we dual-stained kidney sections for both p62 and the lysosome marker, lysosomal-associated membrane protein 1 (LAMP-1), observing no co-localization between the two proteins (**Figure 2F**).

### HDAC6 Inhibition Causes TFEB Acetylation and Nuclear Localization and Attenuates ER Stress Associated Tubule Epithelial Cell Death

**FIGURE 3:**
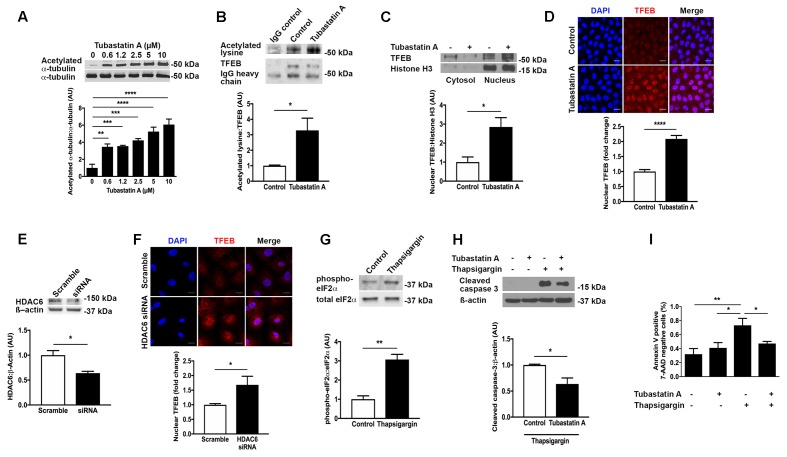
HDAC6 inhibition with Tubastatin A acetylates TFEB, facilitates TFEB nuclear translocation and attenuates programmed cell death in NRK-52E cells. **(A)** Immunoblotting for acetylated α-tubulin in NRK-52E cells treated with Tubastatin A. **(B)** Immunoprecipitation of TFEB and immunoblotting for acetylated lysine in NRK-52E cells treated with Tubastatin A (2.5 μM) for 24 h. **(C)** Immunoblotting for nuclear TFEB in NRK-52E cells treated with Tubastatin A (2.5 μM) for 24 h. **(D)** Immunofluorescence staining for nuclear TFEB in NRK-52E cells treated with Tubastatin A (2.5 μM) for 24 h. **(E)** Knockdown of HDAC6 with short interfering RNA (siRNA) in NRK-52E cells. **(F)** Immunofluorescence staining for nuclear TFEB in NRK-52E cells following HDAC6 knockdown with siRNA. **(G)** Immunoblotting for phosphorylated and total forms of eukaryotic initiation factor 2α (eIF2α) in lysates of NRK-52E cells treated with vehicle or 500 nM thapsigargin for 24 h. **(H)** Immunoblotting for cleaved caspase 3 in NRK-52E cells pretreated with Tubastatin A (2.5 μM) for 4 h followed by thapsigargin (500 nM) for 24 h. **(I)** Flow cytometry for annexin V positive, 7-AAD negative NRK-52E cells pretreated with Tubastatin A (2.5 μM) for 4 h followed by thapsigargin (500 nM) for 24 h. Scale bar = 15 μm. AU, arbitrary units. Values are mean ± SEM. ^∗^*P* < 0.05, ^∗∗^*P* < 0.01, ^∗∗∗^*P* < 0.001, ^∗∗∗∗^*P* < 0.001.

Having observed a reduction in TFEB expression in human and experimental CKD, we set out to explore a therapeutic means of increasing TFEB activity. We speculated that inhibition of the cytosolic deacetylase HDAC6 would affect TFEB acetylation and that TFEB acetylation would in turn influence TFEB nuclear localization. Treatment of proximal tubule lineage NRK-52E cells with the HDAC6 inhibitor Tubastatin A caused a dose-dependent increase in the acetylation of the established HDAC6 substrate α-tubulin (Hubbert et al., 2002) (**Figure 3A**), indicative of HDAC6 inhibition. By immunoprecipitation, we found that in NRK-52E cells TFEB is acetylated under basal conditions and that TFEB acetylation was increased with Tubastatin A (**Figure 3B**). This increase in TFEB acetylation with Tubastatin A coincided with an overall increase in the amount of TFEB present in the cell nucleus (**Figures 3C,D**). Likewise, when we knocked down HDAC6 with short interfering RNA (siRNA) (**Figure 3E**), we also observed an increase in nuclear TFEB (**Figure 3F**). To determine whether TFEB acetylation and nuclear localization affect cell survival in response to ER stress, we exposed NRK-52E cells to the ER stress inducer thapsigargin following pre-treatment with Tubastatin A. First, we confirmed ER stress induction with thapsigargin by immunoblotting cells for p-eIF2a (**Figure 3G**). Either by immunoblotting for cleaved caspase 3 (**Figure 3H**) or by labeling of cells with annexin V and 7-AAD (**Figure 3I**), we found that ER stress induced programmed cell death in NRK-52E cells was significantly diminished with Tubastatin A treatment.

### HDAC6 Inhibition Attenuates Renal Injury in Subtotally Nephrectomized Rats

**FIGURE 4:**
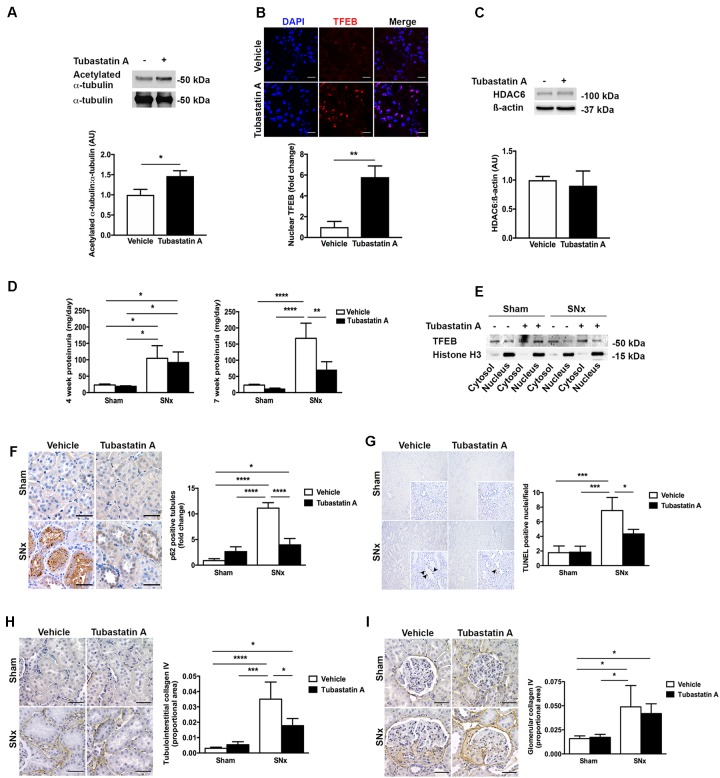
HDAC6 inhibition with Tubastatin A attenuates proteinuria progression and decreases tubule p62, tubule cell death and tubulointerstitial fibrosis in subtotally nephrectomized (SNx) rats. **(A)** Immunoblotting for acetylated α-tubulin in the kidneys of rats treated with Tubastatin A (30 mg/kg thrice weekly for 3 weeks, *n* = 6) or vehicle (*n* = 5). **(B)** Immunofluorescence staining for nuclear TFEB in the kidneys of rats treated with Tubastatin A (*n* = 4) or vehicle (*n* = 5) for 3 weeks. **(C)** Immunoblotting for HDAC6 in the kidneys of rats treated with vehicle (*n* = 3) or Tubastatin A (*n* = 3) for 3 weeks. **(D)** Urinary protein excretion in sham-operated and SNx rats 4 weeks after surgery (before the initiation of treatment) and 7 weeks after surgery (after treatment with Tubastatin A for 3 weeks) (sham + vehicle, *n* = 12; sham + Tubastatin A, *n* = 12; SNx + vehicle, *n* = 8; SNx + Tubastatin A, *n* = 11). **(E)** Immunoblotting for TFEB in cytosolic and nuclear fractions of kidney homogenates from sham and SNx rats treated with vehicle or Tubastatin A. Immunoblot is representative of at least three samples per group. **(F)** Immunohistochemistry for p62 and quantification of tubule p62 immunostaining in kidney tissue from vehicle- or Tubastatin A-treated sham (vehicle, *n* = 10; Tubastatin A, *n* = 10) and SNx (vehicle, *n* = 8; Tubastatin A, *n* = 10) rats. **(G)** TUNEL staining and quantification of tubule TUNEL staining in kidney tissue from vehicle- or Tubastatin A-treated sham (vehicle, *n* = 9; Tubastatin A, *n* = 9) and SNx (vehicle, *n* = 8; Tubastatin A, *n* = 9) rats. Original magnification ×100 (insets [same fields] ×400). The arrows mark TUNEL-positive tubule cell nuclei. **(H)** Immunohistochemistry for collagen IV and quantification of tubulointerstitial collagen IV in kidney tissue from vehicle- or Tubastatin A-treated sham (vehicle, *n* = 10; Tubastatin A, *n* = 10) and SNx (vehicle, *n* = 6; Tubastatin A, *n* = 10) rats. **(I)** Immunohistochemistry for collagen IV and quantification of glomerular collagen IV in kidney tissue from vehicle- or Tubastatin A-treated sham (vehicle, *n* = 10; Tubastatin A, *n* = 10) and SNx (vehicle, *n* = 6; Tubastatin A, *n* = 10) rats. Scale bar = 50 μm, except **(B)** scale bar = 15 μm. AU, arbitrary units. Values are mean ± SEM. ^∗^*P* < 0.05, ^∗∗^*P* < 0.01, ^∗∗∗^*P* < 0.001, ^∗∗∗∗^*P* < 0.001.

**FIGURE 5:**
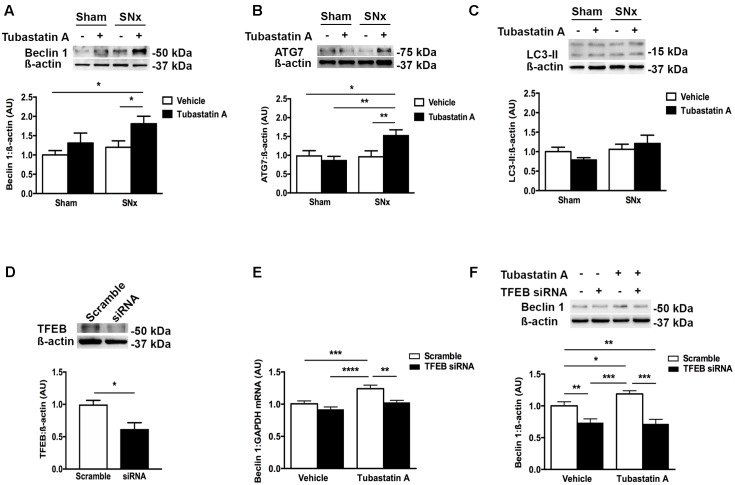
HDAC6 inhibition increases expression of the autophagy markers beclin 1 and ATG7 in subtotally nephrectomized (SNx) rat kidneys and increases beclin 1 expression in a TFEB-dependent manner in NRK-52E cells. **(A–C)** Immunoblotting kidney homogenates for **(A)** beclin 1 (*n* = 4/group), **(B)** ATG7 (sham + vehicle, *n* = 3; sham + Tubastatin A, *n* = 5; SNx + vehicle, *n* = 4; SNx + Tubastatin A, *n* = 4) and **(C)** LC3 (*n* = 5/group). **(D)** Knockdown of TFEB with short interfering RNA (siRNA) in NRK-52E cells. **(E)** Real-time PCR for beclin 1 in NRK-52E cells transfected with TFEB siRNA for 24 h and then treated with Tubastatin A (2.5 μM) for a further 48 h. **(F)** Immunoblotting for beclin 1 in NRK-52E cells transfected with TFEB siRNA for 24 h and then treated with Tubastatin A (2.5 μM) for a further 48 h. Values are mean ± SEM. ^∗^*P* < 0.05, ^∗∗^*P* < 0.01, ^∗∗∗^*P* < 0.001, ^∗∗∗∗^*P* < 0.001.

In our next series of experiments, we set out to determine whether HDAC6 inhibition may affect TFEB nuclear localization and tubule cell injury *in vivo*. We first treated normal rats with Tubastatin A (30 mg/kg thrice weekly s.c.) for 3 weeks, observing a significant increase in renal acetylated α-tubulin levels [indicative of *in vivo* HDAC6 inhibition (**Figure 4A**)] and an increase in renal nuclear TFEB (**Figure 4B**), with no change in renal HDAC6 levels (**Figure 4C**). Next, we randomized rats to undergo sham or subtotal nephrectomy surgery and we followed them for 4 weeks. At this point, animals were stratified by proteinuria (**Figure 4D**) to receive Tubastatin A or vehicle for a further 3 weeks (**Table 1**). Whereas urine protein excretion progressed in vehicle-treated SNx rats, this progression was abated with Tubastatin A treatment (**Figure 4D**). Likewise, hypertrophy of the remnant kidney was also significantly attenuated with HDAC6 inhibition (**Table 1**). Immunoblotting nuclear extracts showed increased TFEB levels with Tubastatin A treatment in sham and SNx rats (**Figure 4E**). In comparison to the kidneys of their vehicle-treated counterparts, the remnant kidney of Tubastatin A treated SNx rats exhibited less tubule p62 immunostaining (**Figure 4F**) and decreased tubule programmed cell death (**Figure 4G**), together with an attenuation in the deposition of collagen IV within the tubulointerstitium (**Figure 4H**). Glomerular collagen IV content, however, was unaffected by Tubastatin A (**Figure 4I**). The attenuation in proteinuria, tubule epithelial cell death and tubulointerstitial collagen IV deposition in SNx rats treated with Tubastatin A coincided with an increase in renal levels of the markers of autophagy induction, beclin 1 (**Figure 5A**) and ATG7 (**Figure 5B**). In contrast, total renal levels of the autophagosome marker microtubule-associated protein light chain 3-II (LC3-II) did not differ between the study groups (**Figure 5C**). Because beclin 1 is a known direct target of TFEB (Palmieri et al., 2011), we finally set out to determine whether HDAC6 inhibition with Tubastatin A increases beclin 1 expression in a TFEB-dependent manner. To address this question, we turned back to our cell culture system and we knocked down TFEB in NRK-52E cells using siRNA (**Figure 5D**). By real-time PCR and immunoblotting, we found that beclin 1 mRNA and protein levels were significantly increased by Tubastatin A and that this increase was negated by TFEB knockdown (**Figures 5E,F**).

**Table 1:** Functional characteristics of sham-operated and subtotally nephrectomized (SNx) rats treated with vehicle or Tubastatin A.

	*n*	Body weight (g)	Left kidney weight (g)	Left kidney weight: body weight (%)	SBP (mmHg)	GFR (ml/min/kg)
Sham + vehicle	12	610 ± 20	1.69 ± 0.06	0.28 ± 0.01	126 ± 2	8.5 ± 0.5
Sham + Tubastatin A	12	562 ± 16^a^	1.56 ± 0.04	0.28 ± 0.01	123 ± 2	8.8 ± 1.0
SNx + vehicle	8	516 ± 11^b^	2.42 ± 0.07^de^	0.47 ± 0.01^de^	204 ± 12^de^	2.8 ± 0.7^de^
SNx + Tubastatin A	11	513 ± 15^bc^	1.86 ± 0.10^fg^	0.36 ± 0.02^deg^	189 ± 12^de^	3.3 ± 0.3^de^

*SBP, systolic blood pressure; GFR, glomerular filtration rate. ^*a*^*p* < 0.05 vs. sham + vehicle, ^*b*^*p* < 0.001 vs. sham + vehicle, ^*c*^*p* < 0.05 vs. sham + Tubastatin A, ^*d*^*p* < 0.0001 vs. sham + vehicle, ^*e*^*p* < 0.0001 vs. sham + Tubastatin A, ^*f*^*p* < 0.01 vs. sham + Tubastatin A, ^*g*^*p* < 0.0001 vs. SNx + vehicle*.

## Discussion

**FIGURE 6:**
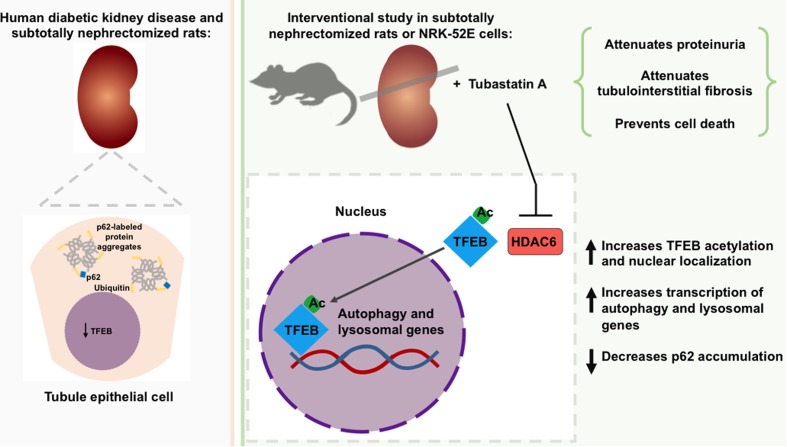
Role of TFEB and of HDAC6 inhibition in the development of chronic kidney disease (CKD). Expression of the transcription factor, TFEB is diminished in the tubule epithelial cells of humans with diabetic kidney disease and subtotally nephrectomized rats and is accompanied by an increase in protein aggregates. Inhibition of the deacetylase HDAC6 increases TFEB acetylation and nuclear localization. In subtotally nephrectomized rats, HDAC6 inhibition attenuates proteinuria, reduces p62 levels, prevents tubule epithelial cell death and attenuates tubulointerstitial fibrosis.

Even though almost half a century has passed since the importance of bulk degradative processes to kidney tubule homeostasis was first postulated (Ericsson, 1969), it is only more recently that these processes have come under the spotlight for their contribution to renal dysfunction and their amenability to therapeutic manipulation (Takabatake et al., 2014). In the present study, we discovered that expression of the transcription factor TFEB, a controller of bulk degradation, is diminished in human diabetic kidney disease and in SNx rats. We observed that HDAC6 deacetylates TFEB and limits its activity by sequestering the transcription factor in the cytosol and that, in SNx rats, HDAC6 inhibition with Tubastatin A attenuated protein aggregation, diminished tubule epithelial cell death, reduced the deposition of fibrotic matrix and prevented proteinuria progression (**Figure 6**). Collectively, these findings (i) demonstrate the occurrence of dysregulated quality control processes in CKD; (ii) identify a regulatory relationship between HDAC6 and TFEB; and (iii) reveal the possible reno-protective benefits of HDAC6 inhibition through enhanced quality control.

Misfolded proteins are sequestered into large protein aggregates within the cytosol by the adaptor protein, p62 through its ubiquitin-associated (UBA) domain and subsequent self-oligomerization through its N-terminal Phox and Bem1p (PB1) domain (Nezis et al., 2008; Komatsu and Ichimura, 2010). Here we found that p62 immunostaining was increased in the tubule epithelial cells of humans with diabetic kidney disease and in SNx rats. In humans we also observed staining for p62 in the nuclei of tubule epithelial cells in kidney sections from individuals with diabetic kidney disease. This observation is consistent with the known nucleoplasmic shuttling of p62 (Pankiv et al., 2010), where it may act as a transcriptional co-activator (Geetha and Wooten, 2002) and it suggests that the role(s) of p62 in CKD may not be solely limited to the organization of misfolded protein aggregates. Indeed, through its interactions with Kelch-like EZH-associated protein 1 (Keap1) and the transcription factor Nrf2, p62 also plays a role in regulating the response to oxidative stress (Komatsu et al., 2010); whereas p62 has also been reported to interact with HDAC6 itself (Yan et al., 2013). Nevertheless, the increase in p62 protein levels determined by immunoblotting in SNx rats and the corresponding increase in protein ubiquitination and eIF2α phosphorylation suggest that increased p62 in kidney disease is at least a partial surrogate for the accumulation of misfolded proteins.

In human diabetic kidney disease and in the kidneys of SNx rats, the increase in protein misfolding coincided with a diminution in the expression of TFEB. Under basal conditions, TFEB is located in the cytosol, where it exists in a phosphorylated state, bound to mTORC1 and the protein complex 14-3-3. Cellular stress inactivates mTORC1, leading to dephosphorylation of TFEB, its release from 14-3-3 proteins and translocation of the transcription factor to the nucleus (Settembre et al., 2011; Martini-Stoica et al., 2016). There, it binds to specific E-box sites of a number of genes that encode proteins important to the autophagy-lysosome pathway (Sardiello et al., 2009; Settembre et al., 2011), ultimately functioning to enhance autophagic flux (Porter et al., 2013). We speculated that HDAC6 may play some role in regulating TFEB activity because of the multiple known roles HDAC6 plays in the autophagy-lysosome pathway (including the cellular localization of other transcription factors) (Lee et al., 2016) and because of the importance of post-translational modifications in regulating TFEB nuclear shuttling. At the early stages of autophagy, HDAC6 helps to organize misfolded proteins in structures known as aggresomes, limiting their ability to impede the cellular machinery and preparing them for engulfment by double-membraned autophagosomes. At the later stages of autophagy, HDAC6 plays an important role in cortactin-mediated fusion of autophagosomes with lysosomes (Lee et al., 2010). Despite these apparent enabling actions, under some circumstances inhibition of HDAC6 has been found to actually promote misfolded protein clearance in certain disease settings (Selenica et al., 2014; Zhang et al., 2014). We wondered whether this may reflect an additional role for HDAC6 in regulating TFEB activity and we found that the HDAC6 inhibitor Tubastatin A increased TFEB acetylation, enhanced the nuclear localization of TFEB and attenuated programmed cell death induced by ER stress. In SNx rats, the reno-protective effects of Tubastatin A were associated with decreased tubule epithelial cell p62 and an upregulation of the TFEB target (Palmieri et al., 2011) and inducer of autophagy, beclin 1 and in cultured cells we found beclin 1 upregulation by Tubastatin A to be TFEB-dependent.

The rational design of Tubastatin A was reported in 2010 (Butler et al., 2010). The structure of the small molecule is typical of that of other HDAC inhibitors that have a wider spectrum of activity, i.e., a zinc-binding group (hydroxamic acid), linker and cap group (Butler et al., 2010). However, the cap group of Tubastatin A was designed to be of sufficient size and rigidity to occupy the comparatively larger catalytic channel rim of HDAC6 but not other HDACs, conferring the small molecule inhibitor with its isoform specificity (Butler et al., 2010). That being said, one study has suggested that Tubastatin A may also have activity against HDAC10 (Oehme et al., 2013). Thus, caution should be taken before ascribing all of the effects of Tubastatin A to the inhibition of HDAC6. Conversely, HDAC6 likely has protein organizing functions that may be uninhibited by blockade of its catalytic activity by Tubastatin A. In particular, whereas HDAC6 possesses two catalytic sites [termed DD1 and DD2, the latter targeted by Tubastatin A (Batchu et al., 2016)], it also possesses a dynein-motor binding domain (Kawaguchi et al., 2003) and a ubiquitin-binding zinc-finger domain (Seigneurin-Berny et al., 2001) that enable it to function as an adaptor protein that organizes p62-labeled proteins for bulk degradation. Nonetheless, the increase in TFEB nuclear localization by either Tubastatin A or HDAC6 siRNA suggest that the nuclear localization of the transcription factor is regulated by the HDAC6 isoform. Likewise, the increase in TFEB acetylation suggests that inhibition of the catalytic activity of HDAC6 by Tubastatin A is responsible for this effect.

Despite the insights gleaned from the current suite of experiments, this study has several limitations and there are several questions that remain unanswered. Firstly, the lysine residue(s) on TFEB that are deacetylated by HDAC6 have not been identified, nor have the mechanisms been resolved by which the acetylation of TFEB by HDAC6 inhibition increases the nuclear localization of TFEB. HDAC6 inhibition has recently been shown to block the interaction between 14-3-3 proteins and their partners (Mortenson et al., 2015) and TFEB is known to associate with 14-3-3 proteins in the cytosol (Martina et al., 2012). Whether (de)acetylation of TFEB affects this interaction and/or whether (de)acetylation of TFEB affects the ability of the transcription factor to bind to its target sites on the genome are unclear. Secondly, in the present study a causative role for TFEB downregulation in misfolded protein accumulation in kidney cells has not been proven. It is also unlikely that enhanced TFEB activity is the sole means by which HDAC6 inhibition may affect renal (patho)physiology. For instance, Tubastatin A reduced renal enlargement in SNx rats, a finding aligned with a recent report of the anti-proliferative effects of HDAC6 inhibition in kidney cells and in cystic kidney disease (Cebotaru et al., 2016). Likewise, we previously reported that pan-HDAC inhibition attenuates renal growth in early experimental diabetes; a finding that we attributed to downregulation of the epidermal growth factor receptor (EGFR) (Gilbert et al., 2011), which may itself be regulated by HDAC6 (Deribe et al., 2009; Gao et al., 2010). Finally, one of the nuances of autophagic processes is their temporality. At the timepoint studied (7 weeks after subtotal nephrectomy surgery and 3 weeks after the initiation of Tubastatin A treatment), we observed an increase in protein levels of the autophagy inducers beclin 1 and ATG7 with Tubastatin A treatment together with a reduction in p62. In cultured cells, we observed that beclin 1 upregulation with Tubastatin A treatment was TFEB dependent. However, the results do not prove a causal association between beclin 1 (or ATG7) upregulation and lower levels of tubule epithelial p62. Indeed, by immunoblotting there was no overall change in the levels of the autophagosome marker LC3-II in kidney homogenates of sham and SNx rats treated with vehicle or Tubastatin A. There are a number of possible explanations for this apparent discordance in renal LC3-II. At a technical level, the findings may indicate the heterogeneous nature of the renal architecture, especially following subtotal nephrectomy surgery. At a biological level, the findings may reflect heightened autophagic flux with HDAC6 inhibition, although this cannot be proven in tissue samples. In spite of these limitations, this study’s findings do provide important new insights, including the downregulation of TFEB in CKD, the functional relationship between HDAC6 inhibition and TFEB activity and the reno-protective effects of HDAC6 inhibition by Tubastatin A.

In summary, the master regulator of the autophagy-lysosome pathway, TFEB is downregulated in CKD accompanied by an accumulation of protein aggregates in tubule epithelial cells. Pharmacological inhibition of the cytosolic deacetylase, HDAC6 hyperacetylates TFEB and causes the transcription factor to localize to the cell nucleus, upregulating the autophagy inducer beclin 1 and attenuating renal decline. Strategies that inhibit the enzymatic actions of HDAC6 and/or enhance the activity of TFEB both warrant further investigation as treatments for CKD.

## Author Contributions

Conceived and designed the experiments: AB, SB, SMa, and AA. Performed the experiments: AB, SB, SMa, TA, KT, SMc, YL, SA, BB, and MK. Contributed the clinical data: LG and FS. Wrote and edited the paper: AB and AA.

## Conflict of Interest Statement

AA has received research support through his institution from AstraZeneca and Boehringer Ingelheim. The other authors declare that the research was conducted in the absence of any commercial or financial relationships that could be construed as a potential conflict of interest.

## Footnotes

1 http://www.ncbi.nlm.nih.gov/tools/primer-blast/
